# Mitochondrial genome diversity and population structure of two western honey bee subspecies in the Republic of South Africa

**DOI:** 10.1038/s41598-018-19759-3

**Published:** 2018-01-22

**Authors:** Amin Eimanifar, Rebecca T. Kimball, Edward L. Braun, James D. Ellis

**Affiliations:** 1 Honey Bee Research and Extension Laboratory, Entomology and Nematology Department, University of Florida, Gainesville, Florida 32611-0620 USA; 2 Department of Biology, University of Florida, Gainesville, Florida 32611 USA

## Abstract

*Apis mellifera capensis* Eschscholtz and *A*.*m*. *scutellata* Lepeletier are subspecies of western honey bees that are indigenous to the Republic of South Africa (RSA). Both subspecies have invasive potential and are organisms of concern for areas outside their native range, though they are important bees to beekeepers, agriculture, and the environment where they are native. The aim of the present study was to examine genetic differentiation among these subspecies and estimate their phylogenetic relationships using complete mitochondrial genomes sequences. We used 25 individuals that were either assigned to one of the subspecies or designated hybrids using morphometric analyses. Phylogenetic analyses of mitogenome sequences by maximum likelihood (ML) and Bayesian inference identified a monophyletic RSA clade, subdivided into two clades. A haplotype network was consistent with the phylogenetic trees. However, members of both subspecies occurred in both clades, indicating that *A*.*m*. *capensis* and *A*.*m*. *scutellata* are neither reciprocally monophyletic nor do they exhibit paraphyly with one subspecies nested within the other subspecies. Furthermore, no mitogenomic features were diagnostic to either subspecies. All bees analyzed from the RSA expressed a substantial level of haplotype diversity (most samples had unique haplotypes) but limited nucleotide diversity. The number of variable codons across protein-coding genes (PCGs) differed among loci, with *CO3* exhibiting the most variation and *ATP6* the least.

## Introduction

The western honeybee, *Apis mellifera* L. (Hymenoptera, Apidae) is a eusocial species and a major pollinator that is ecologically and economically important^1,2^. A global analysis of the modern diversity of *A*. *mellifera* using morphometric and genetic approaches suggests that there are approximately 26 subspecies classified into at least six different evolutionary groups: (A) African subspecies, (M) northern and western European subspecies, (C) north Mediterranean subspecies, (O and Z) Middle Eastern subspecies, and (Y) in Ethiopia^3–7^. This great diversity may have arisen due to regional adaptation to the distinctive ecological conditions in each geographic region^8^.

Eleven *A*. *mellifera* subspecies have been identified throughout Africa based on discrete behavioral, morphological, and regional adaptation to different ecological conditions^9,10^. There are two honey bee subspecies that are indigenous to the Republic of South Africa (RSA), *A*.*m*. *scutellata* Lepeletier 1836 and *A*.*m*. *capensis* Eschscholtz 1821. *Apis mellifera capensis* (the Cape honey bee) is exclusively distributed in the RSA, whereas the distribution of *A*.*m*. *scutellata* extends north, out of the RSA, into the middle of the continent^9^. Both of these subspecies differ reproductively and behaviorally and both exhibit traits that confer strong invasive potential.

*Apis mellifera capensis* can be socially parasitic due to several specific reproductive characteristics that some Cape worker bees exhibit: thelytokous parthenogenesis (the ability of workers to lay diploid, female-destined eggs without mating)^11^, the possession of spermathecae^12^, and high ovariole numbers^13^. Furthermore, Cape honey bee workers have short latency periods before becoming reproductive, and produce queen-like pheromones rapidly after queen loss in the colony^14,15^. In the early 1990’s, *A*.*m*. *capensis* colonies were moved by migratory beekeepers into regions of the RSA where *A*.*m*. *scutellata* was endemic but that historically lacked *A*.*m*. *capensis*^16^. There, *A*.*m*. *capensis* has become a social parasite of *A*.*m*. *scutellata* colonies. This happens when some *A*.*m*. *capensis* worker bees drift into *A*.*m*. *scutellata* colonies and replace the existing queen. The parasitic *A*.*m*. *capensis* worker(s) begin producing offspring, though they cannot reproduce at the rate a normal queen does. Thus, the colony dwindles and ultimately dies. This problem became known as “*capensis* calamity”^17^.

*Apis mellifera scutellata* (the African savannah honey bee) is known for being defensive, having a high swarming rate, being migratory, and possessing other phenotypes many beekeepers view negatively^9,18,19^. This is the bee that was introduced into Brazil in 1957^20,21^ and later became known as the “African,” “Africanized,” or “killer” bee of the Americas as it expanded its range throughout South, Central, and Southern North America^22–24^.

A stable hybrid zone separates the populations of *A*.*m*. *capensis* and *A*.*m*. *scutellata* in the RSA, with *A*.*m*. *scutellata* to the north and *A*.*m*. *capensis* to the south^9^. Neither subspecies has naturally expanded into the other subspecies’ geographic region despite their parasitic (*A*.*m*. *capensis*) or invasive (*A*.*m*. *scutellata*) capabilities^25,26^. In addition to the wide variety of behavioral and reproductive differences between the two subspecies, morphometric comparisons demonstrate that the subspecies have undergone sufficient morphological differentiation to be recognized as distinct subspecies^9^. However, recent studies have revealed multiple diagnostic markers to differentiate different subspecies but *A*.*m*. *capensis* and *A*.*m*. *scutellata* clusters in a single monophyletic RSA clade^27–29^.

Given their unique biologies, it is reasonable to assume that *A*.*m*. *capensis* and *A*.*m*. *scutellata* might have distinct genetic structures. The arrangement of *A*.*m*. *capensis* and *scutellata* based on morphological characters suggests two distinct subspecies, but further examination is required to find reliable and robust genetic markers that are diagnostic for the two subspecies. Given the more rapid evolutionary rate and coalescence of the mitochondrial genome^30–32^, the mitochondrion may yield diagnostic markers even in the absence of diagnostic nuclear markers.

The rapid development of Next Generation Sequencing (NGS)^33,34^ methods has provided opportunities to extract large amounts of data from organisms rapidly, thus improving our understanding of patterns of divergence at multiple taxonomic levels^35,36^. Use of NGS data, combined with the availability of a published mitochondrial genomes makes it possible to assemble the complete mitogenomes of *A*.*m*. *capensis*, *A*.*m*. *scutellata* and hybrid honey bees to examine differentiation among them. Here, we generated complete mitogenome sequences for 25 honey bees sampled from 25 discrete geographical regions across RSA. Given the morphological differentiation between *A*.*m*. *capensis* and *A*.*m*. *scutellata*, we sought to determine if the complete mitogenome sequences could be used to differentiate between the two honey bee subspecies accurately. The results of this study provide new insights regarding the mitogenome diversity of honey bee maternal lineages in RSA, but also clearly underscore the need for large-scale genomic studies in biogeography and evolutionary ecology of *Apis*.

## Results

### Genome structure, organization and composition

We collected complete mitogenomes from 25 honey bees (including two published in^37,38^ (Table 1). The assembled mitogenome sequences of all 25 honey bees were circular molecules that ranged in length from 16,338–16,513 bp (Table 2). On average, we mapped over 70,000 Illumina HiSeq. 3000 paired-end reads (2 × 100 bp) per honey bee sample (range from 33,469–390,359), corresponding to a sequence depth that averaged 479× (range from 204–2,392×). The number of ambiguous sites in final assemblies was low (0.01%). These ambiguous sites were largely in non-coding regions (particularly the AT-rich region). Only one sequence had ambiguities in any of the protein-coding genes (PCGs) or rRNAs. Thus, our results demonstrate the effectiveness of genome skimming for the assembly of complete mitochondrial sequences.

**Table 1:** Summary information for honey bee samples collected in the Republic of South Africa. Bees were sampled from 25 apiaries.

Apiary No.	geographical region	abbreviations	*Apis mellifera* subspecies	geographical coordinates	GenBank Accession Number
1	Bredasdorp	BD	*A*.*m*. *capensis*	34°50ʹS–20°35ʹE	MG552681
2	Citrusdaal	CD	*A*.*m*. *capensis*	32°84ʹS–19°24ʹE	MG552682
3	Cape Town	CT	*A*.*m*. *capensis*	33°96ʹS–18°45ʹE	MG552683
4	George	GE	*A*.*m*. *capensis*	33°98ʹS–22°47ʹE	MG552684
5	Grahamstown	GT	*A*.*m*. *capensis*	33°31ʹS–26°49ʹE	MG552685
6	Knysna	KN	*A*.*m*. *capensis*	34°05ʹS–22°99ʹE	KX870183
7	Langebaan	LA	*A*.*m*. *capensis*	33°00ʹS–18°31ʹE	MG552686
8	Laingsburg	LB	*A*.*m*. *capensis*	33°27ʹS–20°85ʹE	MG552687
9	Moorreesburg	MB	*A*.*m*. *capensis*	33°10ʹS–18°74ʹE	MG552688
10	Modderfontein	MF	*A*.*m*. *capensis*	33°18ʹS–25°80ʹE	MG552689
11	Plettenburg Bay	PB	*A*.*m*. *capensis*	34°05ʹS–23°36ʹE	MG552690
12	Port Elizabeth	PE	*A*.*m*. *capensis*	33°87ʹS–25°39ʹE	MG552691
13	Riversdale	RD	*A*.*m*. *capensis*	34°10ʹS–21°20ʹE	MG552692
14	St. Francis	SF	*A*.*m*. *capensis*	34°17ʹS–24°81ʹE	MG552693
15	Stellenbosch	ST	*A*.*m*. *capensis*	33°85ʹS–18°82ʹE	MG552694
16	Swellendam	SW	*A*.*m*. *capensis*	34°05ʹS–20°65ʹE	MG552695
17	Worcester	WD	*A*.*m*. *capensis*	33°52ʹS–19°49ʹE	MG552696
18	Bloemfontein	BL	*A*.*m*. *scutellata*	29°20ʹS–27°20ʹE	MG552698
19	Kroonstad	KR	*A*.*m*. *scutellata*	27°27ʹS–27°50ʹE	MG552699
20	Pretoria	PT	*A*.*m*. *scutellata*	25°70ʹS–28°10ʹE	MG552700
21	Springbok	SP	*A*.*m*. *scutellata*	29°65ʹS–17°83ʹE	MG552701
22	Upington	UP	*A*.*m*. *scutellata*	28°52ʹS–21°24ʹE	MG552702
23	Vryburg	VR	*A*.*m*. *scutellata*	26°96ʹS–24°76ʹE	MG552703
24	Beaufort West	BW	Hybrid	32°34ʹS–22°62ʹE	KX943034
25	Klawer	KL	Hybrid	32°02ʹS–18°78ʹE	MG552697

Geographical region is identified by the main city/town closest to the sampled apiary and is abbreviated for use elsewhere in the manuscript. Subspecies identity was confirmed morphometrically following^9^. The GPS location of each apiary is noted (geographical coordinates). Finally, we include the GenBank accession numbers for the sequenced mitogenome of each bee.

**Table 2:** Data on the 39 sequenced mitogenomes used in the study.

Gene regions	Aligned length	Parsimony Informative sites	Variable sites	Variable sites (%)	Variable amino acid sites	Variable amino acid sites (%)	Verage AT (%)
*ND2*	1002	23	42	4.2	7	2.1	86.3
*COI*	1566	34	73	4.7	6	1.1	76.1
*CO2*	676	11	30	4.4	3	1.3	80.2
*ATP8*	159	3	7	4.4	1	1.9	88.1
*ATP6*	681	5	13	1.9	2	0.9	84.5
*CO3*	848	19	41	4.8	11	4.2	82.7
*ND3*	354	12	15	4.2	2	1.7	85.5
*ND5*	1668	38	64	3.8	13	2.3	85.5
*ND4*	1319	29	57	4.3	15	3.4	86.5
*ND4L*	264	3	15	5.7	2	2.3	85.9
*ND6*	504	16	27	5.4	5	3.0	86.9
*CYB*	1152	21	42	3.6	7	1.8	80.3
*ND1*	918	21	49	5.3	12	3.9	82.9
tRNAs – 22 genes	1526	14	26	1.7	N/A	N/A	87.0
^*^L-rRNA	1381	17	41	3.0	N/A	N/A	84.3
^†^S-rRNA	787	7	21	2.7	N/A	N/A	81.0
AT-rich region	1053	75	170	16.1	N/A	N/A	96.1
Intergenic sites	982	24	62	6.3	N/A	N/A	94.3
Complete mitogenome	16768	371	789	4.7	86	2.3	84.7

*L-rRNA: Large subunit of ribosomal RNA.

^†^S-rRNA: Small subunit of ribosomal RNA.

Mitogenome content and organization was consistent with published data^37,38^ with 38 regions: 13 protein-coding genes (PCGs), two ribosomal RNAs (lrRNA and srRNA), 22 tRNAs, and an AT-rich non-coding region. The mitochondrial gene orders and arrangements were identical to those of other published *A*. *mellifera* mitochondria^37–40^. The genes encoded on heavy and light strands and the initiation and termination codons for all individuals are identical to those from our previous publications^37,38^. There were not cases where PCGs appeared to have an unusual start or stop codon; all PCGs initiated with ATA, ATC, ATG, or ATT and all ended with TAA or with a T that can be polyadenylated to form a TAA stop codon. All subspecies exhibited the same patterns of start and stop codon usage, such that the same start codon was found in all samples of all subspecies for a given gene. If the T was polyadenylated to form a stop codon, it was the same across all samples.

The nucleotide composition of the mitogenomes of *A*.*m*. *capensis*, *A*.*m*. *scutellata* and hybrids were strongly biased toward A and T with an average AT content of ~85% across the entire genome (Tables 3 and S1). Base composition did vary among regions of the mitochondrial genomes, with the highest AT content being found in the AT-rich region, as expected, and the lowest being found in *COI* (cytochrome oxidase I; Table 2). The number of variable codons differed among the PCGs (Table 2), with Cytochrome c oxidase subunit 3 (*CO3*) exhibiting the highest degree variation (over 4% of amino acids sites varied among samples) while ATP Synthase 6 (*ATP6*) showed much greater conservation (0.9%). Both NADH dehydrogenase 4 (*ND4*) and NADH dehydrogenase 5 (*ND5*) exhibited variation in the number of codons, with most variation observed in *A*.*m*. *capensis* (Table 2). There were no amino acid substitutions that presented synapomorphies uniting any of the subspecies for which multiple individuals were sampled. Instead, substitutions were either found in just a few individuals of a subspecies, were unique to one sample, or were shared among subspecies. The levels of variation differed among gene regions (Table 2), with the PCGs averaging just under 5% of variable sites. While the AT-rich region showed the greatest variation, some of this may be due to errors in alignment since the extreme base composition (over 95% AT) made identification of homology within this region challenging.

**Table 3:** Genome size and nucleotide compositions among the mitogenomes of *Apis mellifera capensis*, *A*.*m*. *scutellata* and hybrid honey bees.

*Apis mellifera* subspecies	whole mtDNA genome		*PCGs		^†^L-rRNA		^∫^S-rRNA		AT-rich region	
	size range and avg bp	AT %	size range and avg bp	AT %	size range and avg bp	AT %	size range and avg bp	AT %	size range and avg bp	AT %
*A*.*m*. *capensis*	16,343–16,513 16,444.9	84.8	3,662–3,665 3,663.1	83.1	1,325–1,328 1,326.7	88	780–786 784.4	81	797–925 862.7	96
*A*.*m*. *scutellata*	16,338–16,479 16,422.4	84.7	3,662–3,663 3,662.1	83.1	1,326–1,328 1,326.4	84.1	785 785	81	767–931 858	96
Hybrids	16,340–16,455 16,397.5	84.7	3,662–3,663 3,662.5	83.1	1,326 1,326	84.1	780–785 782.5	81	798–874 836	95.8

^*^PCGs: protein-coding genes, includes stop codons.

^†^L-rRNA: Large subunit of ribosomal RNA.

^∫^S-rRNA: Small subunit of ribosomal RNA.

When comparing the RSA subspecies and hybrids, there was little variation and the subspecies did not appear distinct in overall characteristics of the mitogenome (Table 3). While the length of the mitogenome, the PCGs, the rRNAs and the AT-rich region varied, this variation occurred within subspecies and did not define subspecies (Table 3).

### Variation among taxa

The multiple sequence alignment of all 39 mitogenomes produced 789 variable sites, of which 371 were parsimony informative. When only the 13 PCGs and two rRNAs were considered, this was reduced to 559 variables sites, of which 278 were parsimony informative. Using the concatenated data set (13 PCGs and two rRNAs), the *A*.*m*. *capensis* mitogenome sequences generated 124 variable positions, of which 44 were parsimony informative. There were 83 variable sites within *A*.*m*. *scutellata* mitogenome sequences, of which 15 were parsimony informative sites (the lower variation in *A*.*m*. *scutellata* is likely due to the smaller number of *A*.*m*. *scutellata* individuals sequenced). The multiple sequence alignment of hybrid mitogenomes included 34 variable sites and no parsimony informative sites.

### Phylogenetic trees and distances among taxa

**Figure 1:**
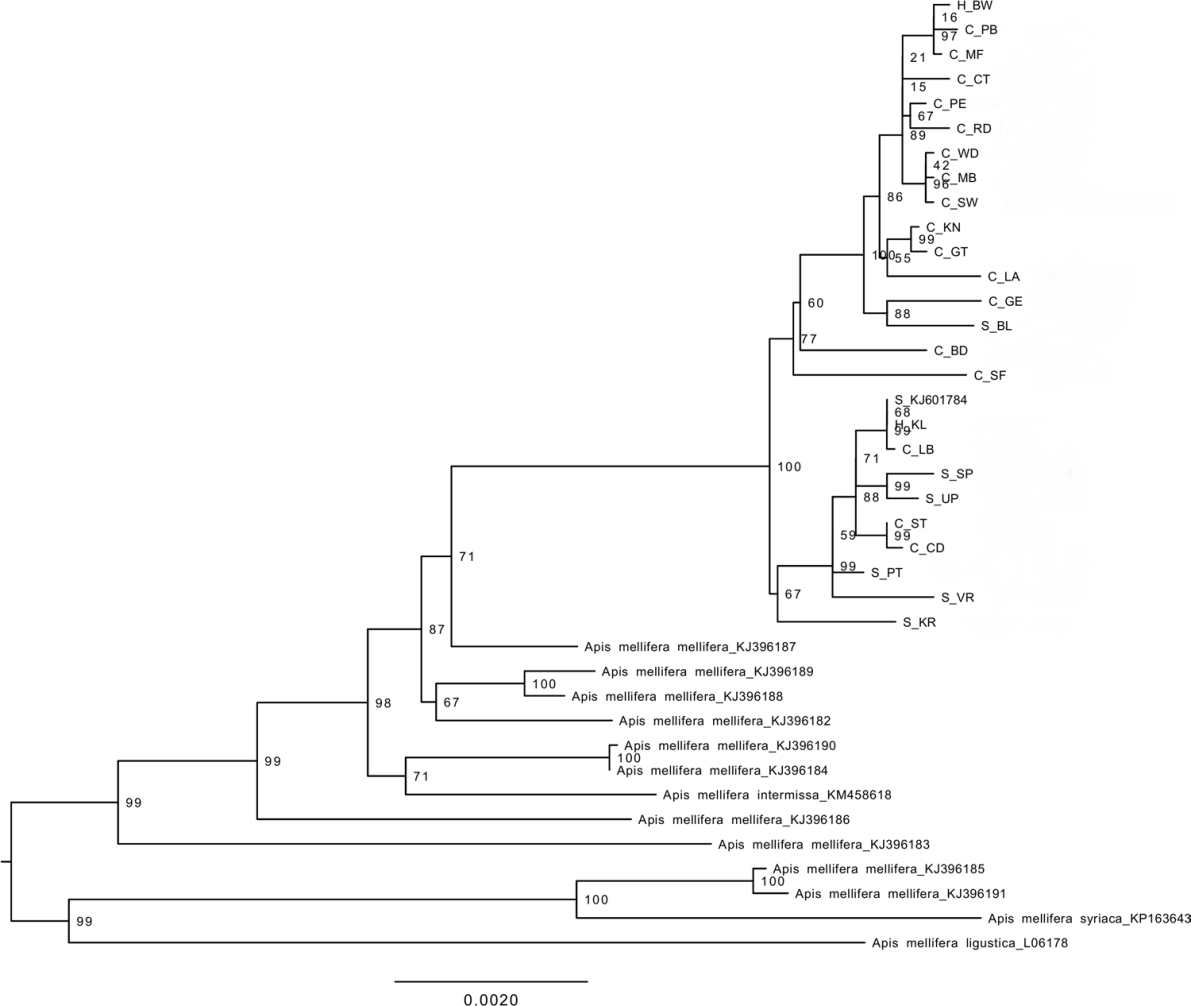
Maximum likelihood phylogenetic tree constructed with RAxML approach for the 39 *Apis mellifera* mitogenomes using concatenated sequences (13 PCGs + two rRNAs). The number represents the bootstrap values which are shown behind each node. The uppermost clade shows the identity of each bee as S for *A*.*m*. *scutellata*, C for *A*.*m*. *capensis*, or H for hybrid. The geographic origin of each bee follows its identity (S, C, or H) and is reported as the abbreviation noted in Table 1.

**Figure 2:**
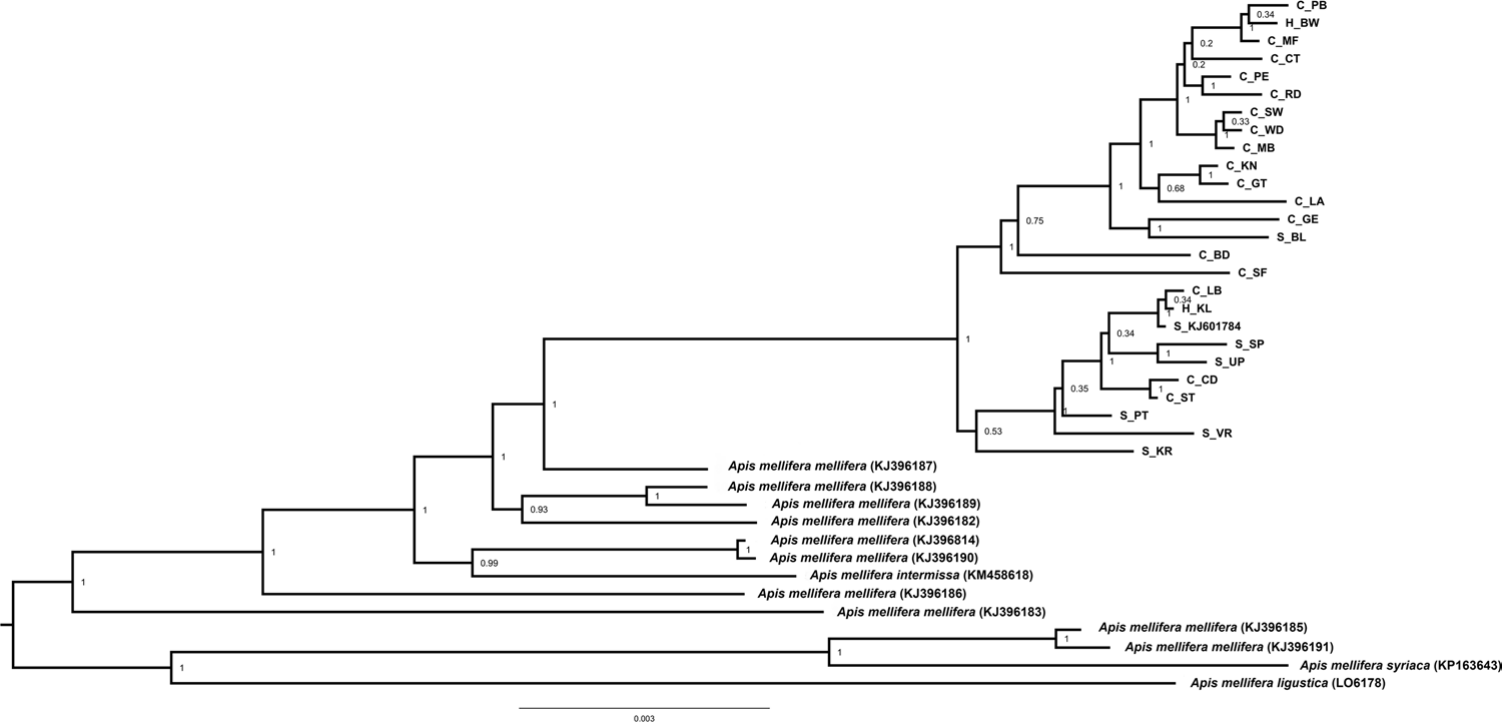
Bayesian inference (BI) phylogenetic tree constructed with MrBayes approach for the 39 *Apis mellifera* mitogenomes using concatenated sequences (13 PCGs + two rRNAs). The number indicates BI posterior probability values which are shown behind each node. The uppermost clade shows the identity of each bee as S for *A*.*m*. *scutellata*, C for *A*.*m*. *capensis*, or H for hybrid. The geographic origin of each bee follows its identity (S, C, or H) and is reported as the abbreviation noted in Table 1.

**Figure 3:**
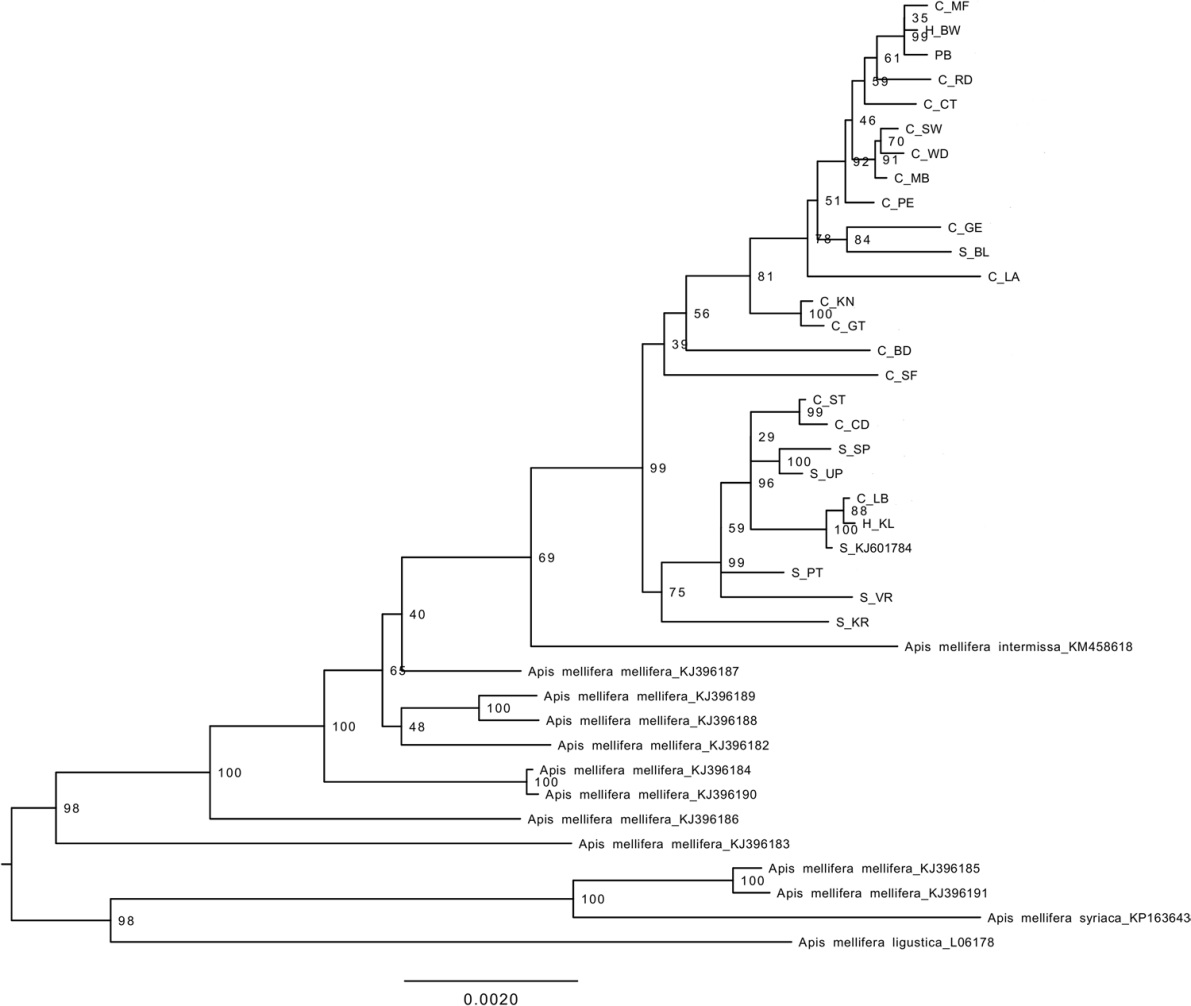
Maximum likelihood phylogenetic tree constructed with RAxML approach for the 39 *Apis mellifera* mitogenomes using complete mitochondrial genome sequences. The number represents the bootstrap values which are shown behind each node. The uppermost clade shows the identity of each bee as S for *A*.*m*. *scutellata*, C for *A*.*m*. *capensis*, or H for hybrid. The geographic origin of each bee follows its identity (S, C, or H) and is reported as the abbreviation noted in Table 1.

**Figure 4:**
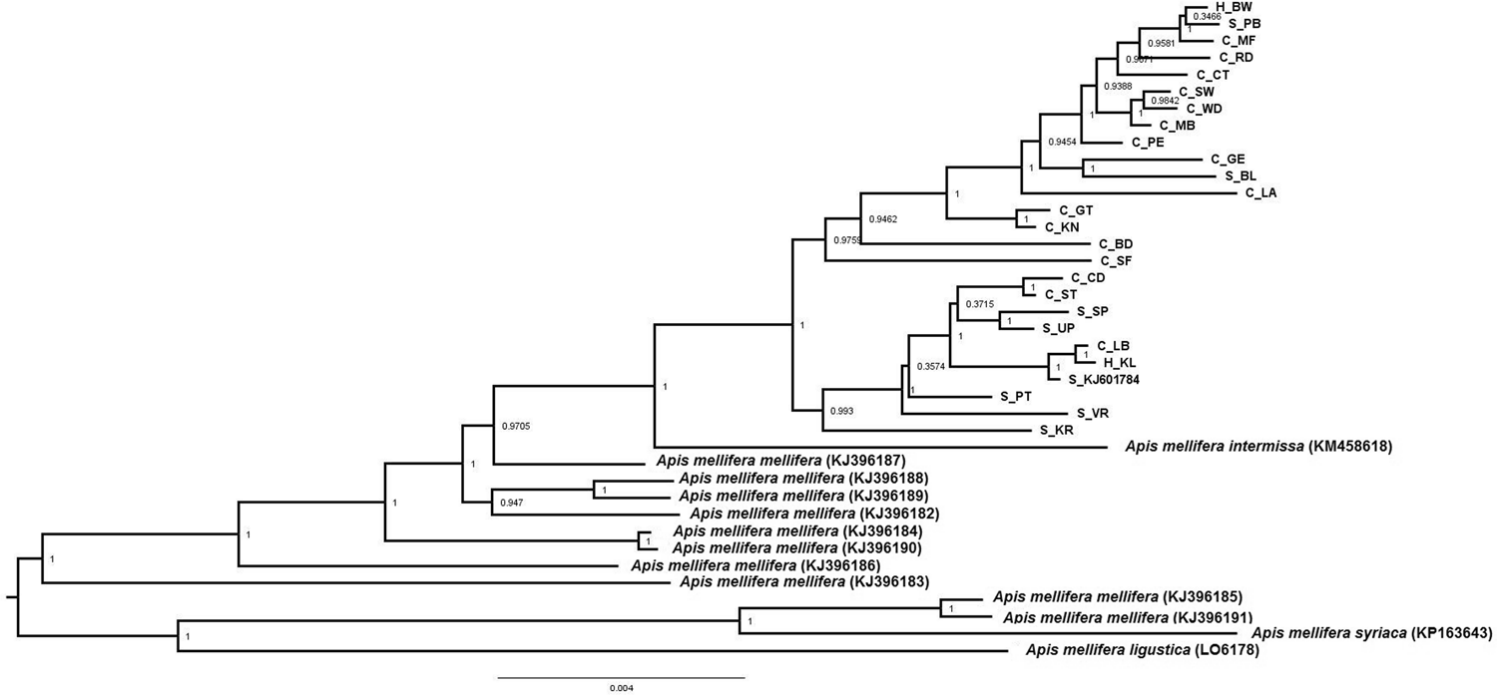
Bayesian inference (BI) phylogenetic tree constructed with MrBayes approach for the 39 *Apis mellifera* mitogenomes using complete mitochondrial genome sequences. The number indicates BI posterior probability values which are shown behind each node. The uppermost clade shows the identity of each bee as S for *A*.*m*. *scutellata*, C for *A*.*m*. *capensis*, or H for hybrid. The geographic origin of each bee follows its identity (S, C, or H) and is reported as the abbreviation noted in Table 1.

The phylogenetic trees using either the complete mitogenome or just the 13 PCGs and two rRNAs showed that the RSA honey bees constituted a well-supported clade, divided into two subclades (Figs 1, 2, 3, and 4). Each subclade consisted of honey bees from different regions and both subspecies. Although this demonstrated a lack of genetic structure between the subspecies, one clade is primarily comprised of *A*.*m*. *capensis* (with only one *A*.*m*. *scutellata*), while the other includes the remaining *A*.*m*. *scutellata* samples (and only three *A*.*m*. *capensis*). Both subclades within the RSA group included one of the hybrid samples. In addition, the other well-sampled subspecies, *A*.*m*. *mellifera*, was also not monophyletic. For both the complete and concatenated datasets, our Bayesian analysis produced a concordant tree topology to the ML tree (Figs 2 and 4).

The two datasets (complete mitogenome or 13 PCGs and two rRNAs) largely yielded similar results (e.g., compare Figs 1 to 2, 3 and 4), except that the placement of *A*.*m*. *intermissa* is in a different phylogenetic position when using the two data sets. *Apis mellifera intermissa* clusters with the RSA clade when complete mitogenomes are analyzed (Figs 3 and 4), whereas it is nested within a larger *A*.*m*. *mellifera* clade when using the concatenated data set (Figs 1 and 2). Thus, the inclusion of the harder-to-align data does have some impact on phylogenetic reconstruction. The inclusion of more sites, especially more variable sites, is expected to result in higher support values. However, there is no consistent pattern with some relationships showing higher support with the complete mitogenome, and others with the concatenated dataset. This may reflect that some sites in the non-coding regions are misaligned, so the increased number of variable sites in complete mitogenome dataset may be offset by greater noise in this alignment. This emphasizes the importance of focusing on regions than can be aligned with confidence.

Using the concatenated data set, the maximum value of the uncorrected genetic distance (*p*-distance) for all pairs of taxa that included one of the 25 RSA mitogenomes was between *A*.*m*. *ligustica* (0.015). The minimum value (0, indicating identical sequences) was only found in nine pairwise comparisons among the 25 South African mitogenomes (data not shown). The overall mean value of pairwise *p*-distance across all 39 mitogenomes was 0.006. The Nei genetic distances within groups of *A*.*m*. *capensis* and *A*.*m*. *scutellata* were 0.002 and 0.001, with an overall distance 0.002.

### Population comparisons and haplotype network

**Figure 5:**
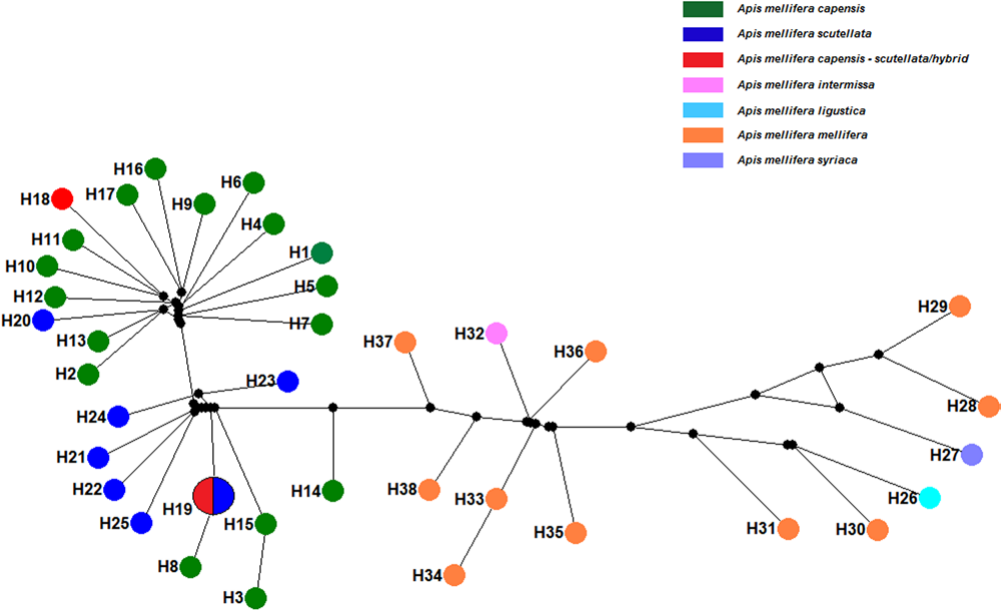
Median-joining parsimony haplotype network constructed for the concatenated sequences of 39 *Apis mellifera* mitogenomes. H1 – H38 refers to the distinct haplotypes. The size of each circle is proportional to haplotype frequency. Each taxa is marked with different colors as shown in the key. H19 was found in two individuals.

The level of haplotype diversity was extremely high for the subspecies for which we had two or more samples. Each honey bee contained a unique haplotype with the exception of samples from hybrid region (KL) and *A*.*m*. *scutellata* (S_NJ601784) which shared a single haplotype (Fig. 5, Table S2). Although haplotype diversity was high among all subspecies, nucleotide diversity was lower among the RSA subspecies (and hybrids) than among *A*.*m*. *mellifera* samples (Table 4). Similarly, the average number of nucleotide differences (K), is much higher for *A*.*m*. *mellifera* than for the RSA subspecies (Table 4). The haplotype network of the concatenated data set generated 38 distinct haplotypes, supporting the results of the phylogeny that showed no population differentiation at the subspecies level (Fig. 5, Table S2).

**Table 4:** Population genetic indices calculated based on concatenated sequences over examined mitogenomes.

*Apis mellifera* subspecies	*N	V	M	H	HD	π	K	Tajima D	Fu’s Fs
*A*.*m*. *capensis*	17	124	125	17	1 ± 0.02	0.002 ± 0.0002	24.1	−1.5–P > 0.1	−4.6–P < 0.05
*A*.*m*. *scutellata*	7	83	84	7	1 ± 0.07	0.002 ± 0.0004	27.1	−1.2–P > 0.1	−0.05–P = 0.5
Hybrids	2	34	34	2	1 ± 0.5	0.002 ± 0.001	34	—	3.5–P = 1
*A*.*m*. *mellifera*	13	424	433	13	1 ± 0.03	0.009 ± 0.001	113	−0.85–P > 0.1	0.064–P = 0.5

*N: number of individuals; V: number of polymorphic sites; M: number of mutations; H: number of haplotypes; HD: haplotype diversity; π: nucleotide diversity; K: average number of nucleotide differences.

## Discussion

In this study, we compared whole mitogenomes from *A*.*m*. *capensis*, *A*.*m*. *scutellata* and hybrids in the RSA, along with published mitogenomes from three other subspecies to infer phylogenetic relationships and genetic differentiation among them. Our phylogenetic analyses suggested that the RSA honey bees are genetically closer to one another than to other taxa included in the tree. Although *A*.*m*. *capensis* and *A*.*m*. *scutellata* are genetically indistinguishable when using mitochondrial genomes, morphological analyses do separate these into two distinct clusters^9^. Furthermore, *A*.*m*. *capensis* and *A*.*m*. *scutellata* have distinctive physiological and behavioral differences^8,9,11^. Thus, the two subspecies are quite distinct and should not be considered as a single subspecies, even if currently no genetic diagnostic markers have been identified for either subspecies.

Although mitogenomes show significant rearrangement of gene orders among Insecta a putative ancestral gene order has been inferred, based in part on studies of Hemiptera^41,42^. There have been rearrangements on the branch leading to *Apis*^42^ but arrangement and gene orders observed in the examined mitogenomes for honey bees from the RSA were identical to those reported for other honey bees, supporting conservation of mitogenome gene order within this genus^40,43,44^. It is possible that there will not be major rearrangements within *Apis* but it is likely that *A*. *mellifera* needs to be studied more broadly across the complete modern diversity of *Apis*^37,38^ to establish this with confidence.

The consensus mitogenomes obtained in this study varied slightly in size: ±170 bp in *A*.*m*. *capensis*; ±141 bp in *A*.*m*. *scutellata;* and ±115 bp for the hybrids. Variation in mitogenome size is typically the consequence of variation in the non-coding regions in insects^45^, which is consistent with our observations of *A*. *mellifera* mitogenomes. Only limited size variation was detected in the coding and rRNA regions. The mitogenomes of *A*. *mellifera* has an AT-bias (84.9%), with guanine as the rarest nucleotide^43^, which is consistent with other observations that the mitochondrial genomes of insects in general have very strong A+T base composition in the non-coding control region^46,47^.

We observed different levels of variation in the PCGs genes across all datasets. The greatest variation looking at nucleotides was in ND4L (followed by ND4), though at the amino acid level the greatest variation was in the *CO3* gene. For both metrics, *ATP6* exhibited the highest level of conservation. De Jager *et al*.^45^ found that the greatest variation in the *Diuraphis noxia* (Hemiptera: Aphididae) was observed in the *ND5* nucleotide gene but the causal effects of variation in *ND5* was not fully understood. These results suggest that the degree of stabilizing selection differs on different genes within the mitochondria, and that this likely also differs among different taxonomic groups. However, too little information currently exists on insect mitochondria to elucidate why there may be different levels of selection among genes, and how universally there are differences among different taxonomic groups.

Using the complete mitochondrial genome sequences, we found that *A*.*m*. *intermissa* clustered at an intermediate position (Figs 3 and 4). The phylogenetic position of *A*.*m*. *intermissa* has been reported by previous investigators and supported by the present study, indicating that *A*.*m*. *intermissa* might be a hybrid subspecies or that the sampled bee was misidentified as a true representative of the subspecies^48^. We support sequencing of additional samples to confirm the phylogenetic position of this subspecies.

Mitochondrial DNA has been widely used as a marker in population genetic, biogeographic, phylogenetic and DNA barcoding studies^49^. Due to their relative rapid coalescence, one should be able to distinguish reciprocally monophyletic groups even when analyses of nuclear markers fail to do so. However, based on our results, mitogenomes appear to be poor diagnostic phylogenetic markers in *Apis*, as has also been found in some other taxa^50^. Our results are not likely due to specific mitochondrial inheritance patterns since similar results have been reported for these subspecies using nuclear SNP loci^27,28^. The absence of genetic differentiation, even with mitochondrial DNA sequences, in taxa where there are clear phenotypic differences among populations has been found in a variety of invertebrate and vertebrate taxa^51–53^, and may be due to the retention of ancestral genetic variation in recently diverged lineages.

An alternative explanation for the lack of genetic differentiation between *A*.*m*. *capensis* and *A*.*m*. *scutellata* could also be due to the impact of beekeeping activities, which can involve the exchange of queens and colonies. These are known to contribute to the admixture pattern among honey bee populations^8,54^. This, in fact, led to a major problem in RSA known as the “*capensis* calamity.” This occurred because *A*.*m*. *capensis* workers were moved from southern portions of RSA into northern portions outside their native range^55^. The *A*.*m*. *capensis* workers became social parasites of *A*.*m*. *scutellata* colonies there, which is a possible reason that their mitogenomes are represented in bees that were morphometrically identified as *A*.*m*. *scutellata*^56^. This problem has slowed or nearly eliminated the movement of managed hives between southern and northern RSA, so the problem should be diminishing. That said, in spite of the movement of bees, the morphological and physiological differences between *A*.*m*. *capensis* and *A*.*m*. *scutellata* persist.

The high genetic diversity in *A*.*m*. *capensis*, *A*.*m*. *scutellata*, hybrids, and other subspecies could be due to large population sizes within their natural habitats^9^. The observed low nucleotide diversity with high haplotype diversity observed in both RSA subspecies might suggest a genetic bottleneck occurred in the past, followed by rapid population expansion for both subspecies. This hypothesis could be investigated via the sampling of more sites and more individuals throughout RSA.

While SNP analyses can differentiate other honey bee subspecies belonging to different evolutionary groups^29,57,58^, mitogenome sequences are not able to differentiate *A*.*m*. *capensis* and *A*.*m*. *scutellata*. There are several reasons this could occur. First, while some hybrids can be identified morphologically, there may be others (particularly if hybrids backcross to one of the parentals) that are morphologically like one subspecies, but may retain the mitogenome of the second subspecies. Second, there could be incomplete lineage sorting, resulting in a mitochondrial gene tree that does not match the subspecies tree^59^. Finally, a small number of genes may be responsible for the phenotypic differences among subspecies, while the remainder of the nuclear genome (as well as the mitogenome) have not yet become reciprocally monophyletic^60^. If this is the case, then the small number of genes responsible for this differentiation would be expected to provide diagnostic markers. Exploration of complete or mostly complete nuclear genomes may be necessary to understand patterns of genetic differentiation, gene flow and divergence among *A*.*m*. *capensis* and *A*.*m*. *scutellata*. Such analyses may eventually identify diagnostic markers, thereby allowing more rapid identification of invasive species. Such methods would enhance our ability to detect hidden genetic structure among regional populations of honey bees in RSA.

## Methods

### Honey bee samples, DNA extraction and sequencing

**Figure 6:**
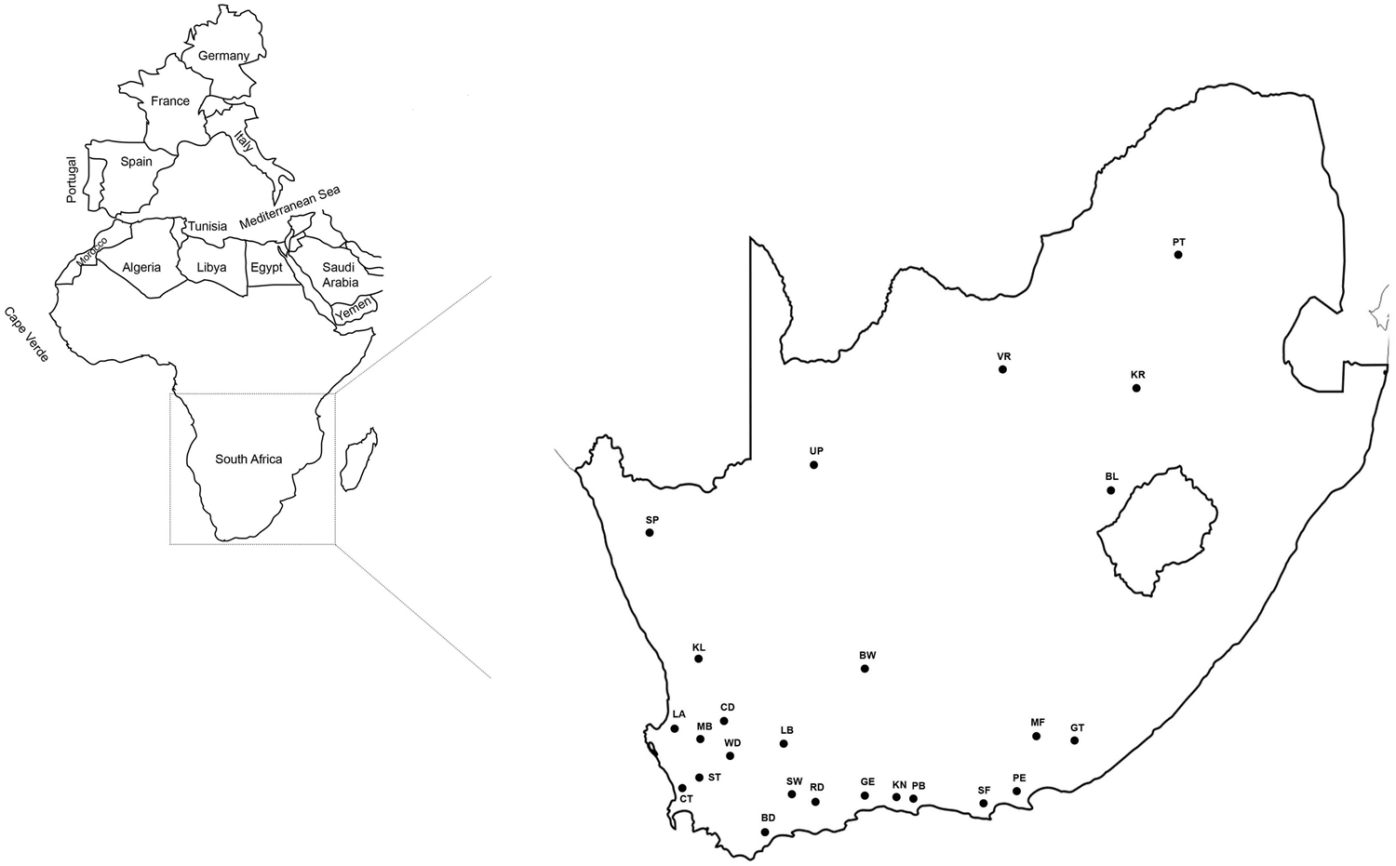
Origin of 25 honey bees collected from managed hives in the Republic of South Africa. The numbers coincide with the sampling regions discussed in Table 1. The map of South Africa was downloaded from this link (http://www.conceptdraw.com/How-To-Guide/geo-map-africa-south-africawas) and re-designed using Adobe Indesign graphical program version CS6 from this link (http://www.adobe.com/products/indesign.html?sdid=KKQLR&mv=search&s_kwcid=AL!3085!3!155829415173!b!!!!indesign%20cs6&ef_id=V2Cx9wAABOu6AgoK:20170830124634:s).

Honey bee samples were collected from managed colonies located across 25 geographical regions in RSA (Table 1, Fig. 6) in April/May 2013 and April 2014^61^. During the field collections, 50–70 adult worker bees were collected from the brood nest of 1–10 colonies located in 1–3 apiaries sampled per each of the 25 geographic regions. In total, 1,000+ colonies were sampled. All bees were collected into 50 ml tubes containing ≥ 98% ethanol. After collection, the samples were imported into the U.S. per USDA APHIS regulations and approval and stored at −80 °C. Bustamante *et al*.^62^ conducted a morphometric analysis on 10 bees from a subset of sampled colonies (N = 240) to classify colonies as *A*.*m*. *capensis*, *A*.*m*. *scutellata*, or hybrids according to standard procedures^9,63^. The thorax of each bee that was identified morphometrically was preserved in 95% ethanol and stored at −80 °C until molecular processing.

One bee from each geographic region was selected for use in the present study. In total, 17 *A*.*m*. *capensis*, six *A*.*m*. *scutellata*, and two hybrid bees were used (Table 1 and Fig. 6), including two whose complete mitogenomes had been published previously^37,38^. Total genomic DNA was extracted from the thoracic tissue of each bee using a Wizard^®^ Genomic DNA Purification kit (Promega, USA) according to the manufacturer’s instructions. The quality of DNA was checked using a 1% agarose gel and quantified using a Quant-iT PicoGreen dsDNA Assay Kit (Life Technologies). From this, 500 ng of DNA was utilized for the construction of paired-end libraries comparable with Illumina sequencing by RAPiD Genomics (Gainesville, Florida, USA). The constructed libraries were multiplexed and sequenced with PE-100 cycle runs (2 × 100 bp) using Next Generation Sequencing on an Illumina HiSeq. 3000 platform (San Diego, California, USA).

### Sequence quality control, assembly and annotation

Mitochondrial data were obtained using the genome skimming method^64^. Short reads were filtered by quality in two steps using the fastx-toolkit (http://hannonlab.cshl.edu/fastx_toolkit/). The first removed bases from the 3′ end of the sequence that had a phred score below 20 and removed the entire read if the resulting length was <50 nt (fastq_quality_trimmer -Q 33 -t 20 -l 50). In the second step, reads that passed the first criteria, but that had ≥10% of the bases with a phred score <20 (fastq_quality_filter -Q 33 -q 20 -p 90) were removed. The resulting sequences were aligned to the bee mitochondria reference genome (NCBI accession NC_0015661) using Mosaik Aligner^65^ version 2.1.33 (parameters -mmp 0.05, -ls 500 -m all -a all).

Illumina sequence reads were mapped to *A*.*m*. *capensis* and *A*.*m*. *scutellata* (KX870183 and KJ601784.1) mitogenomes using Geneious R9.1^66^, and the assembly that exhibited the fewest conflicts (e.g., that mapped to *A*.*m*. *capensis* or to *A*.*m*. *scutellata*) was used. The mapping procedures are described in^37^.

The locations and orientation of protein-coding genes (PCGs), transfer RNA genes (tRNAs) and ribosomal RNA genes (rRNA) genes were identified by multiple alignments to the reference mitogenome using Geneious R9.1^66^. All PCGs for each honey bee mitogenome were translated into amino acids and were manually checked to ensure that each could encode a functional protein (i.g., we examined putative PCGs to determine whether they lacked start or stop codons or exhibited frame shifts and/or premature stop codons.

### Sequence alignment

We aligned 23 novel complete mitogenome sequences with two additional mitogenomes that we published previously^37,38^, as well as 14 published mitogenomes primarily from other *A*. *mellifera* subspecies (Table S3). All mitogenomes were aligned using Muscle 3.8.31^67^ implemented in Mesquite 3.04^68^ with default parameters. The alignment was manually adjusted to maintain reading frame integrity in the protein coding genes.

Our alignment included the complete mitogenome nucleotide sequence. However, since homology was harder to assign in some regions (particularly intergenic regions and the AT-rich region), we also analyzed a concatenated alignment that included all 13 PCGs and two rRNAs to represent a dataset in which we had higher confidence in the alignment. This allowed us to determine if our conclusions depended upon alignment or were robust to inclusion of specific regions and homology assessment in non-coding regions. We estimated *p*-distance between taxa using MEGA7.

### Phylogenetic analyses

Phylogenetic analyses of the complete (entire mitogenome) and concatenated datasets (13 PCGs and two rRNAs) were performed using two different tree-building methods, maximum likelihood (ML) and Bayesian Inference (BI). Maximum Likelihood analyses were performed using RAxML version 8.0.2^69^ with the GTRGAMMA model and 1,000 rapid bootstraps to assess nodal support. BI was performed based on the concatenated data set using MrBayes version 3.2.4^70^. Before running MrBayes, the complete and concatenated datasets were run through jModelTest 3.7^71^ and the AIC was used to select the best model. The best model for the complete dataset was GTR+I+G (shape = 0.7050, pinvar = 0.81), and the best model for the 13 PCGs and two rRNAs was TVM+I+G (shape = 0.8920, pinvar = 0.834). The BI used two independent runs with 8,000,000 generations in each and four chains. Each chain was sampled every 1,000 generations with a burn-in of 25%. The remaining trees were condensed using a 50% majority-rule consensus tree with posterior probabilities.

We also assessed the evolutionary distance between *A*.*m*. *capensis* and *A*.*m*. *scutellata* using uncorrected *p*-distances among unique haplotypes of the 13 PCG and two rRNA data set with MEGA7^72^.

### Population genetic analyses

Our initial results suggested there could be some bias due to mis-alignment in the analysis of the complete dataset. Therefore, we focused population genetic analyses on the 13 PCGs and two rRNA dataset. A haplotype network was generated using the median-joining method with default settings in NETWORK v. 4.6.1.3^73^ for the concatenated dataset (13 PCGs and two rRNAs) from the 39 samples (25 sequenced from the RSA, and 14 previously published mitogenomes from Africa and Europe). All networks were visualized and manually adjusted to ensure sufficient resolution. We computed the haplotype diversity (HD), nucleotide diversity (π), number of haplotypes (H), number of variable sites (V), number of mutations (M), average number of nucleotide differences (K) and neutrality indices using DnaSP v5^74^.

## Electronic supplementary material

Supplementary Tables
