# Supplementary material for: Mitochondrial genome diversity and population structure of two western honey bee subspecies in the Republic of South Africa
Source: Sci Rep. 2018 Jan 22;8:1333. doi: 10.1038/s41598-018-19759-3 (PMC5778041; doi:10.1038/s41598-018-19759-3)
Supplement: Supplementary file 1 — Supplementary Tables [file 41598_2018_19759_MOESM1_ESM.doc]

**Mitochondrial genome diversity and population structure of two western honey bee subspecies in the Republic of South Africa**

Amin Eimanifar^1*^, Rebecca T. Kimball^2^, Edward L. Braun^2^ & James D. Ellis^1^

^1^Honey Bee Research and Extension Laboratory, Entomology and Nematology Department, University of Florida, Gainesville, Florida, 32611-0620, USA

^2^Department of Biology, University of Florida, Gainesville, Florida, 32611, USA

^1*^Corresponding author, Phone: 352 273 3958; (Fax): 352 392 0190

E-mail: [amineimanifar1979@gmail.com](mailto:amineimanifar1979@gmail.com)

**Supplementary information**

**Supplementary Table S1.** Genome size and nucleotide compositions among the mitogenomes of *Apis mellifera capensis*, *A.m. scutellata* and hybrid honey bees. The region’s abbreviation corresponds to the regions described in Table 1 and Fig. 6.

**Supplementary Table S2.** Data matrix containing 38 unique haplotypes with their frequencies among 39 *Apis mellifera* using 574 bp of the concatenated (13 PCGs + two rRNAs) sequences. H = haplotype, F = haplotype frequency. The region’s abbreviations correspond to those listed in Table 1.

**Supplementary Table S3**. Information on the 14 additional (i.e. those other than *Apis mellifera scutellata* and *A.m. capensis*) mitogenomes used in the study. The subspecies identity, Gen Bank accession number, and reference in which the mitogenome announcement was made are listed for each sample.

**Supplementary Table S1**

| **apiary abbreviation (subspecies of bee present)** | **whole mtDNA genome** | **^*^PCGs** | **^†^L-rRNA** | **^∫^S-rRNA** | **AT-rich region** |
| --- | --- | --- | --- | --- | --- |
|  | **Size (bp) AT %** | **Size (bp) AT %** | **Size (bp) AT %** | **Size (bp) AT%** | **Size (bp) AT %** |
| BD (*A.m. capensis*) | 16,467 84.8 | 11,032 83.2 | 1,327 84 | 785 81 | 886 95.9 |
| CD (*A.m. capensis*) | 16,447 84.7 | 11,032 83.1 | 1,326 84.1 | 785 81 | 883 96 |
| CT (*A.m. capensis*) | 16,442 84.7 | 11,032 83.1 | 1,326 84.2 | 785 81 | 859 96 |
| GE (*A.m. capensis*) | 16,493 84.2 | 11,032 83.1 | 1,325 84.2 | 786 81 | 908 96.2 |
| GT (*A.m. capensis*) | 16,428 84.7 | 11,032 83.1 | 1,327 84.3 | 785 81 | 850 96.2 |
| KN (*A.m. capensis*) | 16,470 84.7 | 11,032 83.1 | 1,326 84.2 | 785 81 | 881 96.1 |
| LA (*A.m. capensis*) | 16,473 84.8 | 11,032 83.1 | 1,328 84.2 | 785 81 | 886 96 |
| LB (*A.m. capensis*) | 16,468 84.7 | 11,032 83 | 1,327 84.2 | 785 80.9 | 881 95.9 |
| MB (*A.m. capensis*) | 16,453 84.8 | 11,032 83.1 | 1,327 84.2 | 785 81 | 861 95.9 |
| MF (*A.m. capensis*) | 16,463 84.8 | 11,032 83.1 | 1,327 84.2 | 785 81 | 874 96.1 |
| PB (*A.m. capensis*) | 16,466 84.8 | 11,032 83.1 | 1,327 84.2 | 778 80.8 | 886 96 |
| PE (*A.m. capensis*) | 16,515 84.8 | 11,032 83.1 | 1,327 84.2 | 785 81 | 925 96.2 |
| RD (*A.m. capensis*) | 16,482 84.5 | 11,032 83.1 | 1,326 84.2 | 785 81.1 | 894 95.9 |
| SF (*A.m. capensis*) | 16,467 84.8 | 11,032 83.2 | 1,327 84.1 | 785 81 | 888 95.9 |
| ST (*A.m. capensis*) | 16,423 84.2 | 11,032 83 | 1,326 84.1 | 785 81 | 868 95.9 |
| SW (*A.m. capensis*) | 16,439 84.7 | 11,032 83.1 | 1,327 84.3 | 785 81 | 858 96 |
| WD (*A.m. capensis*) | 16,459 84.8 | 11,032 83.1 | 1,327 84.2 | 785 81 | 870 96 |
| BL (*A.m. scutellata*) | 16,479 84.9 | 11,032 83.2 | 1,347 84.3 | 785 81 | 933 96.2 |
| KR (*A.m. scutellata*) | 16,454 84.8 | 11,032 83.1 | 1,326 84.1 | 785 80.9 | 893 96.3 |
| PT (*A.m. scutellata*) | 16,462 84.8 | 11,032 83 | 1,326 84.1 | 785 81 | 879 96.2 |
| SP (*A.m. scutellata*) | 16,450 84.7 | 11,032 83 | 1,326 84.1 | 785 81 | 869 95.9 |
| UP (*A.m. scutellata*) | 16,364 84.7 | 11,032 83.1 | 1,326 84.1 | 785 81.3 | 797 95.7 |
| VR (*A.m. scutellata*) | 16,339 84.6 | 11,032 83 | 1,326 84.2 | 785 81 | 768 95.8 |
| BW (hybrid) | 16,340 84.7 | 11,032 83.1 | 1,326 84.2 | 785 81 | 793 95.6 |
| KL (hybrid) | 16,469 84.6 | 11,032 83 | 1,326 84.1 | 785 80.9 | 889 96 |
| ^*^PCGs: protein-coding genes; ^†^L-rRNA: Large subunit of ribosomal RNA; ^∫^S-rRNA: Small subunit of ribosomal RNA | | | | | |

**Supplementary Table S2**

| **H** | **F** | ***Apis mellifera* subspecies** | **geographical regions** |
| --- | --- | --- | --- |
| H1 | 1 | *A.m. capensis* | KN |
| H2 | 1 | *A.m. capensis* | BD |
| H3 | 1 | *A.m. capensis* | CD |
| H4 | 1 | *A.m. capensis* | CT |
| H5 | 1 | *A.m. capensis* | GE |
| H6 | 1 | *A.m. capensis* | GT |
| H7 | 1 | *A.m. capensis* | LA |
| H8 | 1 | *A.m. capensis* | LB |
| H9 | 1 | *A.m. capensis* | MB |
| H10 | 1 | *A.m. capensis* | MF |
| H11 | 1 | *A.m. capensis* | PB |
| H12 | 1 | *A.m. capensis* | PE |
| H13 | 1 | *A.m. capensis* | RD |
| H14 | 1 | *A.m. capensis* | SF |
| H15 | 1 | *A.m. capensis* | ST |
| H16 | 1 | *A.m. capensis* | SW |
| H17 | 1 | *A.m. capensis* | WD |
| H18 | 1 | Hybrid | BW |
| H19 | 2 | Hybrid &  *A.m. scutellata* (S_NJ601784) | KL |
| H20 | 1 | *A.m. scutellata* | BL |
| H21 | 1 | *A.m. scutellata* | KR |
| H22 | 1 | *A.m. scutellata* | PT |
| H23 | 1 | *A.m. scutellata* | SP |
| H24 | 1 | *A.m. scutellata* | UP |
| H25 | 1 | *A.m. scutellata* | VR |
| H26 | 1 | *A.m. ligustica* (L06178) | - |
| H27 | 1 | *A.m. syriaca* (KP163643) | - |
| H28 | 1 | *A.m. mellifera* (KJ396185) | - |
| H29 | 1 | *A.m. mellifera* (KJ396191) | - |
| H30 | 1 | *A.m. mellifera* (KJ396183) | - |
| H31 | 1 | *A.m. mellifera* (KJ396186) | - |
| H32 | 1 | *A.m. intermissa* (KM458618) | - |
| H33 | 1 | *A.m. mellifera* (KJ396184) | - |
| H34 | 1 | *A.m. mellifera* (KJ396190) | - |
| H35 | 1 | *A.m. mellifera* (KJ396182) | - |
| H36 | 1 | *A.m. mellifera* (KJ396187) | - |
| H37 | 1 | *A.m. mellifera* (KJ396188) | - |
| H38 | 1 | *A.m. mellifera* (KJ396189) | - |

**Supplementary Table S3**

| **no.** | ***Apis* subspecies** | **GenBank accession numbers** | **References** |
| --- | --- | --- | --- |
| 1 | *Apis mellifera ligustica* | L06178 | 43 |
| 2 | *Apis mellifera syriaca* | KP163643 | 44 |
| 3 | *Apis mellifera intermissa* | KM458618 | 40 |
| 4 | *Apis mellifera scutellata* | KJ601784 | 39 |
| 5 | *Apis mellifera mellifera* | KJ396185 | 75 |
| 6 | *Apis mellifera mellifera* | KJ396191 | 75 |
| 7 | *Apis mellifera mellifera* | KJ396183 | 75 |
| 8 | *Apis mellifera mellifera* | KJ396186 | 75 |
| 9 | *Apis mellifera mellifera* | KJ396184 | 75 |
| 10 | *Apis mellifera mellifera* | KJ396190 | 75 |
| 11 | *Apis mellifera mellifera* | KJ396182 | 75 |
| 12 | *Apis mellifera mellifera* | KJ396187 | 75 |
| 13 | *Apis mellifera mellifera* | KJ396188 | 75 |
| 14 | *Apis mellifera mellifera* | KJ396189 | 75 |

75. Fuller, Z. L. *et al*. Genome-wide analysis of signatures of selection in populations of African honey bees (*Apis mellifera*) using new web-based tools. *BMC Genomics* **16**(1), (2015).
